# The effect of nurse-led bladder training on urinary tract outcomes after kidney transplantation

**DOI:** 10.3389/fneph.2026.1830698

**Published:** 2026-07-29

**Authors:** G. Albertema, A.C.P. Boskma, B.H. Meppelink, J.M. Vonk, S.J.L. Bakker, A. Ozyilmaz, M.F.C De Jong

**Affiliations:** 1 Department of Geriatrics, University Medical Center Groningen, Groningen, Netherlands; 2 Department of Nephrology, University Medical Center Groningen, Groningen, Netherlands; 3 Cross Connection Patient Care, University Medical Center Groningen, Groningen, Netherlands; 4 Department of Cardiology, Amsterdam University Medical Center, Amsterdam, Netherlands; 5 Department of Epidemiology, University of Groningen, University Medical Center Groningen, Groningen, Netherlands

**Keywords:** bladder training, kidney transplant patients, kidney transplantation, nurse-led interventions, urinary tract outcomes

## Abstract

**Introduction:**

Optimal bladder function is crucial for kidney transplant success. Bladder training could help prevent dysfunction and complications, yet evidence-based guidelines for bladder training methods are limited. This study evaluates two protocols: standard hourly training versus tailored to preoperative urine output, assessing urinary tract outcomes.

**Methods:**

This pre-post study used data from the TransplantLines data and biobank on kidney transplant patients admitted in 2019 (standard bladder training) and 2022 (new protocol) to a Dutch academic hospital. Standard bladder training involved hourly urination with regular post-void residual checks, whereas the new protocol adjusted urination frequency and scan schedules based on preoperative urine output. Logistic regression analyses were performed to evaluate the following outcomes: residual urine volume > 100ml, urinary tract infection or re-catheterization within three months post-transplantation.

**Results:**

The analysis included 211 patients (70% male, mean age 55, n=100 standard protocol, n=111 new protocol). Multivariate analysis showed no differences between the protocols in the investigated outcomes. A significant interaction between protocol and age on re-catheterization was found suggesting higher re-catheterization risk in older patients under the new protocol.

**Discussion:**

The revised protocol might promote patient well-being and simultaneously improve nurse working conditions.

## Introduction

The global burden of chronic kidney disease (CKD) is substantial and growing, approximately 10% of adults worldwide are affected by some type of CKD, resulting in 1.2 million deaths yearly (1–3). Kidney transplantation (KT) is a critical intervention for patients with end-stage renal disease, significantly reducing morbidity, mortality, costs, and improving quality of life (4). One of the important elements in KT care is maintaining optimal bladder function, which is essential for transplant success and patient well-being (5–7).

Bladder training can be defined as ‘a nurse-led intervention aimed at improving bladder function through education and scheduled voiding intervals’. It aims to correct maladaptive voiding patterns, enhance control over urgency, increase bladder capacity, and reduce incontinence (8). Although its importance in preventing post-transplant bladder dysfunction is recognized (6, 7), evidence-based guidelines remain limited (9–11). Most studies concern other patient groups (12, 13), or lack comprehensive outcomes (11), and findings are inconsistent, with little research specific to kidney transplant recipients (KTR) (14). When studies do address transplantation care, interventions focus on paediatric populations (15–17), on the preoperative phase (18), or on medication treatment (19). Moreover, based on a national Dutch inquiry, variation in bladder training protocols was identified across transplant centers, with no standardized guidelines or supporting evidence (20). Approaches differ in timing, frequency of voiding, and monitoring methods, ranging from individualized protocols based on urine output to fixed schedules (20). Techniques to assess bladder retention also vary, as do nighttime voiding strategies (20).

Bladder training before hospital discharge seems particularly crucial for KTR due to a decreased bladder capacity, and in case of anuria a defunctionalized bladder, anatomical changes following the transplantation, and an aging population undergoing KT. Post-operative bladder training is thought to help reduce these risks by restoring bladder function and ensuring proper urine flow, thereby protecting the transplanted kidney. Given the variety in bladder training protocols, optimization of the bladder training based on scientific research may improve urinary tract outcomes.

This research gap presents a challenge to the development of evidence-based protocols that are tailored to the needs of KTR. To address this gap, the present study aims to evaluate the effect of bladder training tailored to preoperative 24-hour urine production on post-void residual volume, the incidence of urinary tract infections (UTIs) and incidence of re-catheterization in adult KTR, compared to standard bladder training with hourly daytime and bi-hourly nighttime bladder emptying. While the primary aim of this study was to evaluate and improve clinical outcomes, the revised protocol has been shown to effectively promote patient well-being, especially sleep quality, and simultaneously improves nurse working conditions through streamlined care practices and fewer unnecessary interruptions (14). By combining a brief exploratory literature search in PubMed, the national Dutch inquiry, and evaluation of protocol changes, this study contributes to evidence-based nursing care and supports a more efficient, patient-centered approach in the post-transplantation setting.

## Patients and methods

### Study design

In the University Medical Center Groningen (UMCG) the standard protocol was used until 2019. Thereafter in 2020 the revised protocol was developed and implemented in 2021. In this study we compared outcomes between subjects receiving a KT in 2019 and 2022.

### Study population

In the UMCG, approximately n=170 KTs are performed annually. The population of our study cohort consisted of adult patients who received a KT. Patients included in the TransplantLines data and biobank cohort study (21, 22) and admitted to the transplantation ward in 2019 and 2022 of the UMCG for a KT surgery were eligible for inclusion. Patients who received a combined kidney-pancreas transplant were excluded, as well as those who had pre-existing urogenital issues before surgery (e.g., self-catheterization, Bricker ileal conduit, Benigne Prostatic Hyperplasia). No formal *a priori* power calculation was performed, as this is a retrospective study with a fixed sample size (23). The numbers of KTR were 153 and 190 in 2019 and 2022, respectively.

### Data collection

Data was collected from KTR admitted in 2019 and 2022. Data was retrieved from the TransplantLines database as well as the electronic patient records of patients eligible for inclusion. Primary outcomes, all documented by physicians and nurses, were: (1) urine residual > 100 milliliter (ml, after removing catheter, based on local guidelines, less than 100 ml is the value at which discharge from the hospital is allowed), (2) whether a UTI occurred within three months post-transplant (A urinary tract infection was diagnosed if there was a positive culture, antibiotic treatment or a physician’s diagnosis), and (3) whether re-catheterization was needed during three months after the operation. Re-catheterisation was defined as the reinsertion of an indwelling catheter following its removal on day 5, or the use of intermittent catheterisation. Outcome (1) urine residual was measured only after the catheter had been removed. Outcomes (2) UTI and (3) re-catheterisation were measured throughout the full three-month follow-up period. Additionally, baseline characteristics were extracted, including (a) patient number, (b) measurement moment (pre= 2019 and post= 2022), (c) age in years at the time of transplantation, (d) gender (male/female), (e) dialysis status, (f) diabetic medication, (g) type of donor (living or post-mortal) and (h) pre-operative urine production in 24hours in ml. These variables were selected by consensus within the research team, as they are considered potential determinants of the outcome based on existing literature and clinical relevance (24–26).

### Intervention

Before the protocol modification, bladder training was conducted as follows: on day 5 after surgery, the bladder catheter was removed in the morning, and the patient was instructed to urinate every hour during the day and every two hours at night. In the morning and evening a bladder scan was performed. If the post-void residual volume exceeded 150 ml, the attending physician was consulted.

Initially, we attempted to base a revised bladder training protocol on existing literature. However, no suitable evidence-based insights were available. PubMed was searched (and updated in December 2025) with the following search string: (“Kidney Transplantation”[Mesh] OR kidney transplant*[tiab] OR renal transplant*[tiab]) AND (“Urinary Catheterization”[Mesh] OR “Urinary Catheters”[Mesh] OR “Catheters, Indwelling”[Mesh] OR catheter*[tiab]) AND (“Urinary Bladder”[Mesh] OR bladder[tiab]) AND (training*[tiab] OR retrain*[tiab] OR intervention*[tiab] OR bladder function*[tiab] OR rehabilitation*[tiab] OR restorat*[tiab]) NOT (“Child”[Mesh] NOT “Adult”[Mesh]). The search retrieved n=37 studies, of which n=33 were excluded based on title and n=4 on full-text screening by GA and AB. Exclusion criteria were population and/or intervention not suitable (**Supplementary Material 1**).

**Figure 1:**
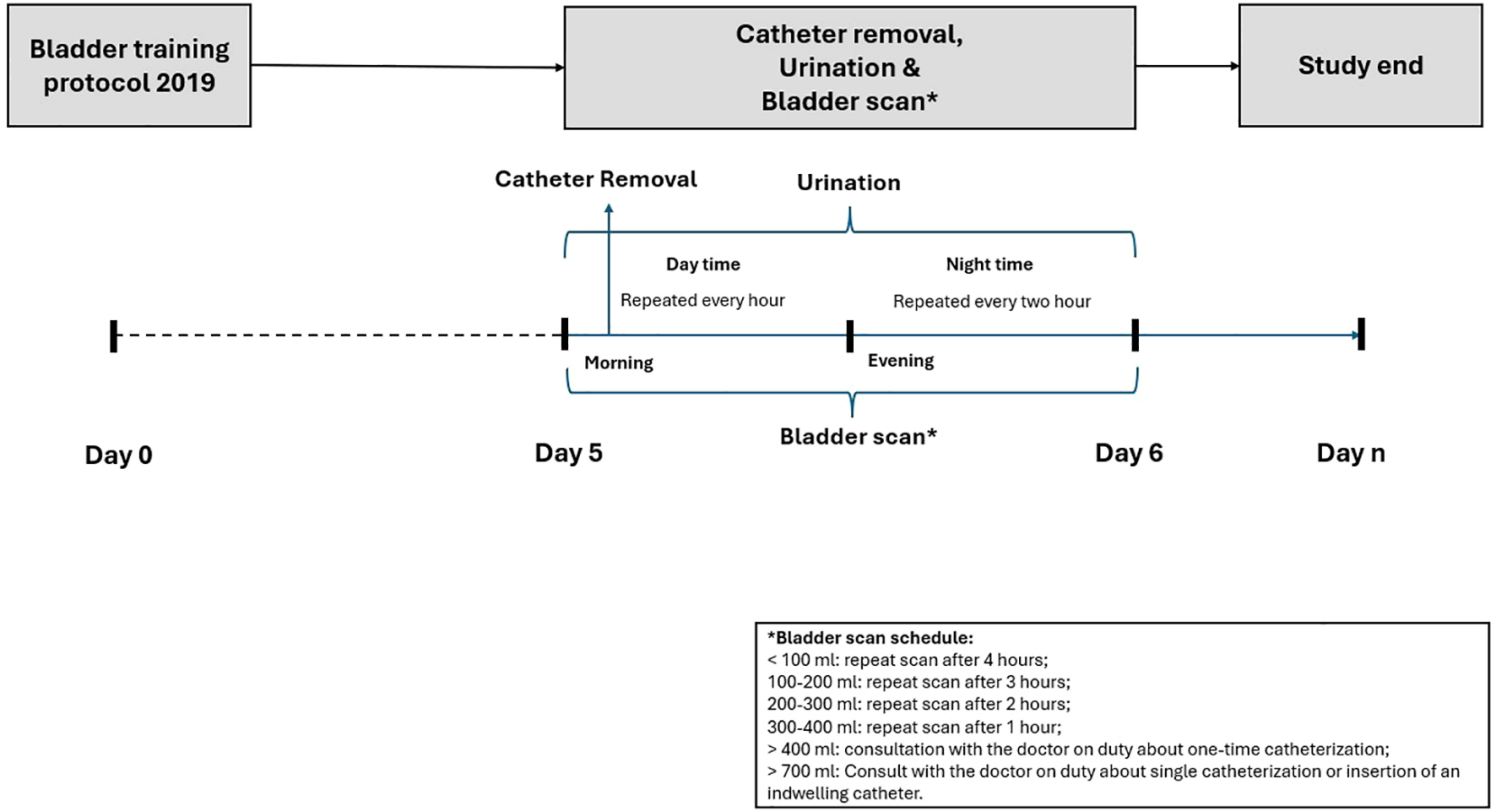
Bladder training protocol 2019.

**Figure 2:**
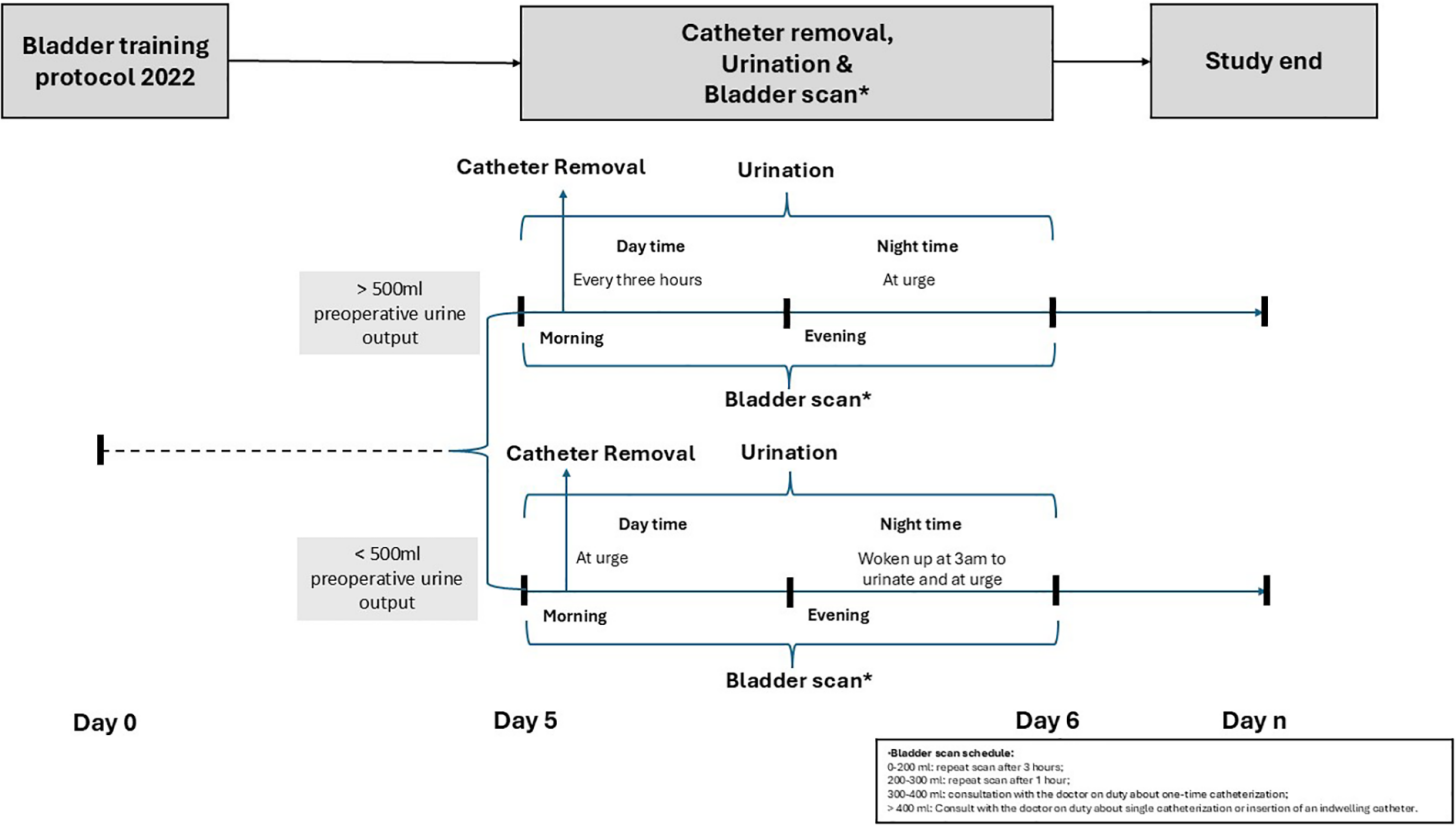
Bladder training protocol 2022.

Therefore, a new protocol was ultimately developed in 2020–2021 by the local kidney transplantation team, drawing on clinical expertise. The aim was to create a protocol that would be less burdensome for both the patient and the nursing staff, while still ensuring optimal outcomes for KTR. The adjustments were carefully considered to balance the needs of the patient with the operational realities of care, ultimately leading to a more efficient and patient-friendly approach. Thus, in the new bladder training protocol, a distinction was made based on whether patients had pre-operative urine production in 24 hours more or less than 500 ml. The cut-off value of 500 ml was selected after discussion among the clinical experts within the research team and based on local practice, expected bladder capacity, degree of pre-transplant urinary output, and experience with defunctionalized bladder. In the new protocol patients with pre-operative urine production more than 500 ml are instructed to urinate every three hours during the day with two scheduled bladder scans and urinate as needed at night. Patients with pre-operative urine production less than 500 ml are instructed to urinate as needed during the day with two scheduled bladder scans and to be awakened once at night to urinate. For patients with preoperative urine output of < 500 ml, the number of mandatory urination moments during the day was reduced from 14 times to 0 times, whereby the patient was allowed to urinate at their own discretion, and from 7 times to once during the night. For patients with preoperative urine output of > 500 ml, the reduction was from 14 times to approximately 5 times during the day, and from 7 times to 0 times during the night. In addition, the bladder scan schedule has been specifically adjusted for KTR. The main changes concern the volume thresholds: the volume for repeating a bladder scan after 1 hour has been reduced from 300–400 ml to 200–300 ml, and the threshold for placing an indwelling catheter has been lowered from a maximum of 700 ml to 400 ml. For a schematic presentation see **Figures 1**, **2**. The complete description of the protocols are included in **Supplementary Material 2**.

### Ethics approval and consent to participate

This study was part of the TransplantLines biobank (NCT03272841). In this biobank, all (potential) solid organ transplantation patients and (potential) living organ donors (aged> 18 years) of the UMCG were invited to participate. All participants gave written informed consent to participate TransplantLines biobank, while additional consent for the present retrospective analysis was not required according to local legislation and institutional requirements. Ethical approval has been granted to the authors prior to the start of study by the authorized Medical Ethics Research committee of Groningen (register number: 2014/077). The study adheres to the UMCG Biobank Regulation and is in accordance with the WMA Declaration of Helsinki and the Declaration of Istanbul. The study was conducted according to the principles of General Data Protection Regulation (AVG).

### Statistical analysis

Descriptive statistics were performed to describe the samples. Chi-square tests were used to compare baseline characteristics between the pre- and post-implementation groups to assess group comparability. Since age is a continuous variable a t-test was conducted.

Univariable and multivariable logistic regression analyses were conducted for each of the three primary outcomes post-void urine residual volume > 100 ml, UTIs and re-catheterization. In these univariable analyses the effect of the new bladder training protocol compared to the former protocol was assessed, as were the associations of age, gender, donor type (living vs. post-mortal) and preoperative urine output (≤500 ml vs. >500 ml) with the outcomes. Odds ratios with corresponding 95% confidence intervals were reported to estimate the strength and direction of associations.

In the multivariable analyses, the effect of the new bladder training protocol compared to the old protocol was assessed with adjustment for the mentioned covariates.

Subsequently, to investigate if the effect of the new bladder training protocol was dependent on age, gender, donor type and pre-operative urine output, interaction terms were added to the univariable regression models (i.e. models included the intervention, the covariate, and the interaction between intervention and covariate). Interactions were analysed in an exploratory manner; therefore, the results should be interpreted as hypothesis-generating. P-values less than 0.10 were considered statistically significant for interactions due to the small sample size (27).

Finally, the significant interactions were added to the multivariable regression models. Missing data were handled by excluding observations with missing values from the analysis. The Strengthening the Reporting of Observational Studies in Epidemiology (STROBE) was used to facilitate reporting and visualising the results (28).

## Results

### Sample descriptions

**Figure 3:**
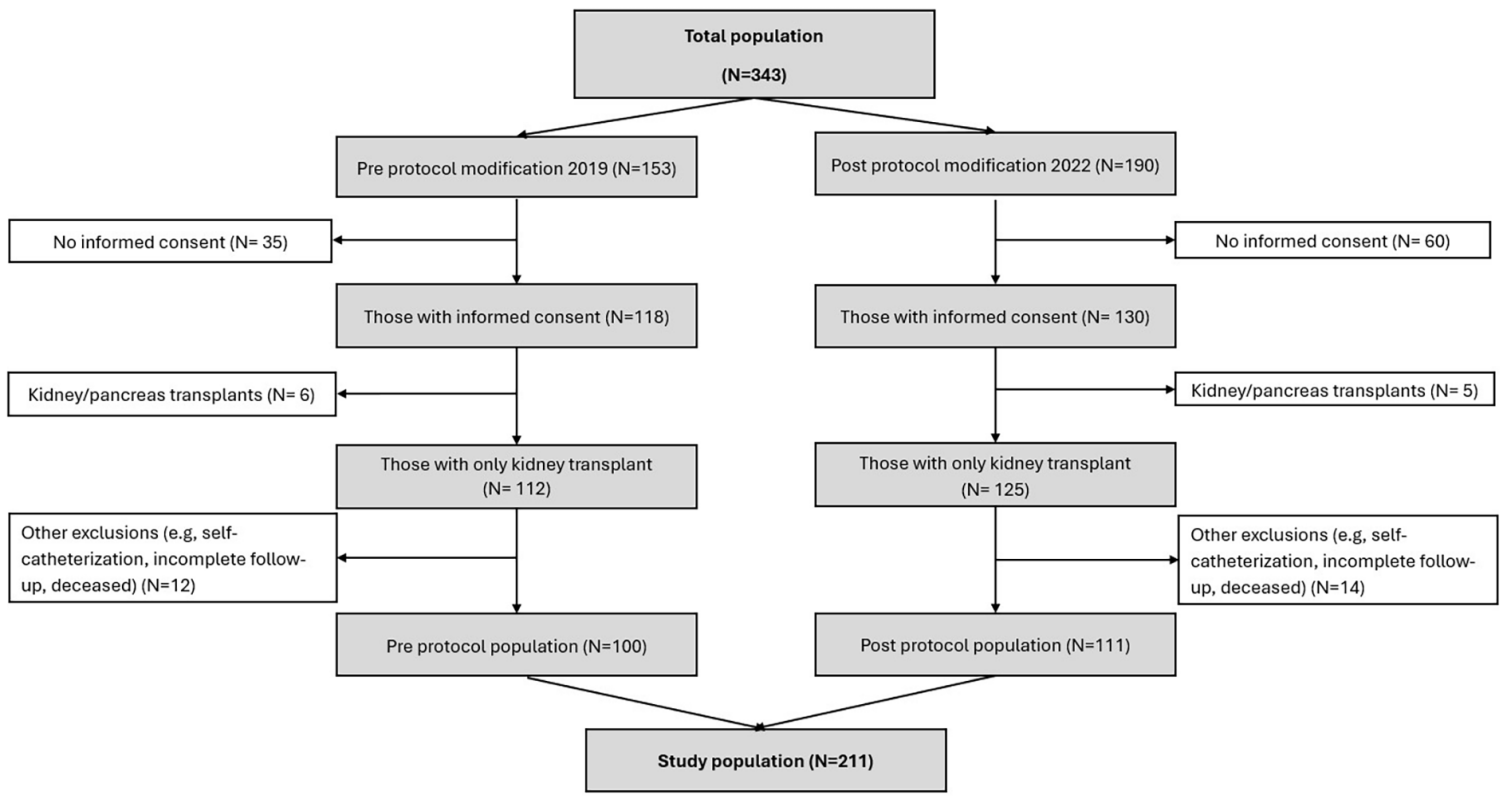
Included patients in the analysis.

Of the 153 patients in 2019 and 190 patients in 2022, in total 211 (61.5%) were included in the study. Eleven patients were excluded because of a simultaneous kidney-pancreas transplantation (n=6 in 2019 and n=5 in 2022), and 26 patients were excluded because of pre-existing urogenital issues before the surgery (e.g., self-catheterization, Bricker ileal conduit), incomplete follow-ups, and death (in total n=12 in 2019 and n=14 in 2022). Additionally, 95 patients were not included since informed consent for the TransplantLines data and biobank cohort was not obtained (n=35 in 2019 and n=60 in 2022). Reasons for not willing to participate were not registered. Finally, a total of 100 patients in 2019 and 111 patients in 2022 were included in the analysis (**Figure 3**).

**Table 1** shows the baseline characteristics of the included patients pre and post protocol modification. Gender distribution was similar between groups, with males comprising approximately 69% pre-protocol and 71.2% post-protocol (p = 0.73). The mean age did not differ significantly (54.9 years pre vs. 56.0 years post; p = 0.46). Preoperative urinary output categories (≤500 ml vs. >500 ml) were comparable between groups (27.6% vs. 28.8% ≤500 ml; p = 0.84). A statistically significant difference was observed in donor type distribution (p = 0.05), with a lower proportion of postmortal donors in the pre-protocol group (30%) compared to the post-protocol group (43%). Regarding clinical outcomes, the post-void urine residual volume (>100 ml in 16% pre vs. 19% post; p = 0.61), incidence of UTIs (15% pre vs. 14% post; p = 0.76) and re-catheterization rates (11% pre vs. 9% post; p = 0.63) showed no significant differences between groups.

**Table 1:** Baseline characteristics per measurement moment.

Baseline characteristics	Pre protocol modification (2019)	Post protocol modification (2022)	P-value
Included transplanted patients (n)	**100**	**111**	
Age (years range)	18-79	22-82	0.46
Age (Mean, (SD))	54.9 (14.6)	56.0 (15.6)	
Gender			0.73
Male (n (%))	69 (69%)	79 (71%)	
Female (n (%))	31 (31%)	32 (29%)	
Dialysis status			
Haemodialysis (n (%))	40 (40%)	44 (39%)	0.96
Peritoneal dialysis (n (%))	19 (19%)	13 (12%)	0.14
Pre-emptive (n (%))	41 (41%)	54 (49%)	0.27
Use of diabetes medication (yes n (%))	15 (15%)	14 (13%)	0.62
Donor type			**0.05**
Living (n (%))	70 (70%)	63 (57%)	
post-mortal (n (%))	30 (30%)	48 (43%)	
Preoperative 24h urine volume			0.84
500 mL or less (n (%))	27 (28%)	32 (29%)	
More than 500 mL (n (%))	71 (72%) 2missing	79 (71%)	
Urine residual in milliliter			0.61
More than 100 (n (%))	16 (16%)	20 (19%)	
100 or less (n (%))	84 (84%)	87 (81%) 4 missing	
Urinary tract infection			0.76
Yes (n (%))	15 (15%)	15 (14%)	
Re-catheterization			0.63
Yes (n (%))	11 (11%)	10 (9%)	

bold = p < 0.05.

**Table 2** presents results of the logistic regression analyses.

**Table 2:** Univariate and multivariate logistic regression, including interaction term, for estimating the effect of the protocol adjustments on different patient outcomes.

Results	Urine Residual > 100 ml	Urinary Tract Infection	Re-catheterization
	OR (95% CI)(P-value)	OR (95% CI) (P-value)	OR (95% CI) (P-value)
Univariate ()			
Post versus pre *	1.21 (0.59-2.49) (0.61)	0.89 (0.41-1.92) (0.76)	0.80 (0.33-1.98) (0.63)
Age	1.02 (1.00-1.05) (0.07)	1.02 (0.99-1.05) (0.17)	1.03 (1.00-1.07) (0.07)
Female gender	0.64 (0.27-1.49) (0.30)	1.70 (0.76-3.78) (0.19)	0.52 (0.17-1.62) (0.26)
Post-mortal donor	**2.64 (1.27-5.48) (0.01)**	1.60 (0.73-3.49) (0.24)	**2.51 (1.00-6.25) (0.05)**
Preoperative up >500ml	0.81 (0.37-1.78) (0.60)	0.90 (0.39-2.11) (0.82)	0.61 (0.24-1.55) (0.30)
Multivariate			
Post versus pre*	1.16 (0.54-2.48) (0.71)	0.85 (0.38-1.89) (0.69)	0.71 (0.28- 1.80) (0.47)
Mean Age	1.01 (0.99- 1.04) (0.35)	1.02 (0.99-1.05) (0.30)	1.03 (0.99- 1.07) (0.19)
Female gender	0.50 (0.20- 1.24) (0.14)	1.66 (0.74-3.74) (0.22)	0.47 (0.15-1.50) (0.20)
Post-mortal donor	2.23 (0.96- 5.21) (0.06)	1.34 (0.55- 3.28) (0.53)	2.00 (0.70- 5.72) (0.20)
Preoperative up >500ml	1.04 (0.45- 2.40) (0.93)	0.99 (0.40-2.42) (0.98)	0.77 (0.28-2.09) (0.61)
Multivariate with interaction**			
Post versus pre*	1.16 (0.54-2.48) (0.71)	0.73 (0.31-1.70) (0.46)	0.43 (0.13-1.44) (0.17)
Mean Age		1.05 (0.99-1.11) (0.09)	1.08 (1.00-1.18) (0.07)
Female gender		1.71 (0.75-3.86) (0.20)	0.48 (0.15-1.55) (0.22)
Post-mortal donor		1.31 (0.52-3.28) (0.57)	1.84 (0.62-5.49) (0.28)
Preoperative up >500ml		0.96 (0.39-2.35) (0.92)	0.73 (0.27-1.99) (0.54)

bold = p < 0.05.

Univariate model: each covariate is included separately in the models (i.e. five models). Multivariate model: all covariates are included simultaneously in one model.

Number of events:.

Urine Residual: Pre protocol modification > 100 ml: 16 (16%).

Urine Residual: Post protocol modification > 100 ml: 20 (19%).

Urinary Tract Infection: Pre protocol modification: 15 (15%).

Urinary Tract Infection: Post protocol modification: 15 (14%).

Re- catheterization: Pre protocol modification: 11 (11%).

Re- catheterization: Post protocol modification: 10 (9%).

Odds ratios are reported for the category coded as 1, with the category coded as 0 serving as the reference.

*Post versus pre: pre = 0 and post = 1.

**outcome urine residual > 100 ml no significant interactions.

### Urine residual

Implementation of the new protocol did not significantly change the occurrence of urine residual >100 ml, no significant difference was found in multivariate analysis, and no interaction terms showed p-values <0.10 (**Supplementary Material 3**). Post-mortem donation was the only covariate significantly associated with a higher occurrence of urine residual >100 ml (p = 0.009).

### Urinary tract infection

The revised protocol, age, gender, donor type, and preoperative urine output were not significantly associated with UTI risk, and multivariate analysis showed no difference between pre- and post-protocol implementation. A univariate model suggested an interaction between protocol effect and age (p = 0.06) (**Supplementary Material 3**), but this was not significant in the multivariate model.

### Re-catheterization

The new protocol did not significantly affect re-catheterization, and multivariate analysis confirmed no difference between pre- and post-implementation. Post-mortem donation showed a borderline association (OR = 2.51, p = 0.05). Age interacted with the new protocol in the univariate analysis (OR = 1.09, p = 0.05), and this interaction remained significant in the multivariate model (p = 0.04), suggesting higher re-catheterization risk in older patients under the new protocol (**Supplementary Material 3**).

## Discussion

This study examined the effect of bladder training tailored to preoperative urine production (≤ or > 500 mL in 24 hours) on > 100 ml urine residual incidence, UTI’s, and the frequency of re-catheterization in adult KTR. In this single-centre pre-post cohort, a urine-output-tailored bladder training protocol was not associated with a statistically significant increase in post-void residual urine, UTI, or re-catheterisation within three months. These results are reassuring for maintaining the quality and safety of post operative kidney transplant care. While not measured, the revised protocol hypothesizes improvements in patient well-being, especially sleep quality, and could simultaneously enhance nurses’ working conditions through streamlined care practices and fewer unnecessary interruptions. Larger prospective studies incorporating patient-reported outcomes and staff feedback are needed.

After implementation of the new protocol, there was a modest increase in the proportion of patients with post-void residual urine volume >100ml, a likely consequence of reduced voiding frequency and longer bladder filling times. However, this increase did not translate into higher re-catheterization rates. In this study, a cut-off value of 100 ml was applied, in accordance with local guidelines stipulating patients may be discharged if two consecutive bladder scan measurements show residual volumes less than 100 ml. Given the small decrease in re-catheterizations alongside the small increase in residual volume, a greater proportion of patients in the post-implementation group might be expected to have residual volumes between 100 and 300 ml, as catheterization is initiated from 300 ml. In practice, however, this was not convincingly observed, and the observed decrease in re-catheterizations may plausibly be explained by other factors, such as dislodgement of a double-J stent and self-catheterization, rather than by the protocol itself, suggesting that any increase in residual volumes cannot be conclusively attributed to the intervention.

Donor type was identified as factor influencing the occurrence of urine residual and re-catheterizations, which is understandable as recipients of post-mortal KT generally present with higher risk (29), and the post-implementation group contained a significantly greater proportion of these recipients. Patients receiving a post-mortal KT are typically on dialysis for a longer period, resulting in a smaller bladder capacity and, consequently, a higher likelihood of residual urine (30). Preoperative voiding data confirm this, showing that recipients of post-mortal kidneys more often produced less than 500 mL of urine preoperatively and exhibited higher residual volumes post-operatively. This pattern confirms donor type is an important confounder in interpreting post-operative bladder function outcomes. Because living donors, more common in the pre-protocol group, are linked to better outcomes, the protocol’s effect may be underestimated, as this group already had a more favourable prognosis. Nevertheless, causal conclusions are limited because the study does not adjust for potential mediators or confounders such as cold ischemia time, catheter duration, dialysis duration, and duration of anuria.

Beyond functional outcomes, the findings are highly relevant from a clinical and policy making perspective. While not measured in this study, the revised protocol presumed the benefits of patient well-being, especially sleep quality, and simultaneously improves nurse working conditions through streamlined care practices and fewer unnecessary interruptions, as patients are asked to void less frequently, thereby requiring less assistance from nursing staff and fewer bladder scans. These effects are meaningful given that workload and patient sleep quality are well-known challenges in hospital care (31, 32). In the context of increasing healthcare demands and a declining workforce, reducing workload without compromising quality or safety of care is of considerable value (33). Furthermore, previous research has shown that patients’ sleep is frequently disturbed by alarms or other patients, and poor sleep can impair recovery (31). By reducing the need for nighttime interventions, the new protocol is assumed to support both patient rest and the rest of other patients in shared rooms, potentially contributing to improved recovery times. Before and during the implementation of the new protocol, nurses were informed of the new standard by training sessions and reminded daily by the coordinating nurse. Although adherence to the protocol was not measured, it is plausible that nurses applied the intervention uniformly because patient enrolment began after six months of working daily with the new protocol on the wards.

To our knowledge, this is the first study to evaluate bladder training in a clinical transplant setting. The new protocol is specifically tailored to the unique needs of kidney transplant patients, enhancing both patient-friendliness and procedural efficiency. The involvement of a multidisciplinary team in the study design and interpretation of results further strengthens the findings, as diverse perspectives and end-user input help bridge the gap between research and clinical practice.

However, several limitations must be acknowledged. The selective sampling strategy may introduce bias, limiting the generalizability of the results to a broader population and higher risk recipients. Exclusion of patients with pre-existing urogenital conditions, incomplete follow-ups, and death could overlook important variations in outcomes and may have introduced selection bias. Follow-up was limited to three months post-transplant, which may not fully capture the long-term effects of the protocol. Additionally, a larger sample size would be beneficial to confirm the observed results and ensure their robustness. Finally, the interaction analyses should be interpreted with caution given the wide confidence intervals and the fact that adherence was not measured is a limitation.

The findings of this study highlight the need to evaluate current post-operative bladder training practices in clinical settings. Research on this topic across diverse patient populations remains limited, and these results may stimulate further investigation by demonstrating that alternative approaches can be implemented in a manner that benefits both patients and nursing staff without compromising the quality or safety of care. Future studies on post-operative bladder training should account for patient-specific factors such as preoperative bladder capacity, baseline urine output, comorbid urogenital conditions, and age, as younger patients appear to experience better outcomes. Further research could explore optimal cut-off values for different age groups, which may inform whether age-specific guidelines are warranted and where these thresholds should be set. Implementation in a multidisciplinary context is recommended, ideally incorporating patient perspectives to optimize adherence and satisfaction. Although the experiences of healthcare professionals were not assessed in this study, including their feedback in future research would provide important insights into feasibility and workflow impact. Moreover, a prospective design allows for stronger causal inferences regarding the impact of the protocol.

## Conclusion

In this single-centre pre-post cohort, the revised protocol was not associated with a statistically significant increase in post-void residual urine, UTI, or re-catheterisation within three months. Hence, these results did not indicate deterioration in these quality indicators of post-operative kidney transplant care. While not measured, the revised protocol hypothesizes improvements in patient well-being, especially sleep quality, and could simultaneously enhance nurses’ working conditions through streamlined care practices and fewer unnecessary interruptions. These findings provide guidance for clinicians, nurses, leaders and researchers. Larger prospective studies incorporating patient-reported outcomes and staff feedback are needed. Future research should focus on optimizing protocols for different age groups and patient populations to further improve value-based healthcare and effectiveness.

## Data Availability

The original contributions presented in the study are included in the article/**Supplementary Material**. Further inquiries can be directed to the corresponding author.
