# Supplementary material for: The effect of nurse-led bladder training on urinary tract outcomes after kidney transplantation
Source: Front Nephrol. 2026 Jul 29;6:1830698. doi: 10.3389/fneph.2026.1830698 (PMC13461544; doi:10.3389/fneph.2026.1830698)
Supplement: Supplementary file 1 [file Table1.docx]

**Supplemental material**

**Supplemental file 1	Studies literature review**

| **First Author** | **Year** | **Title** | **Screening on title** | **Screening on full-text** |
| --- | --- | --- | --- | --- |
| Leslie | 2024 | Vesicoureteral Reflux | INS |  |
| Alghoraibi | 2023 | Recurrent Urinary Tract Infection in Adult Patients, Risk Factors, and Efficacy of Low Dose Prophylactic Antibiotics Therapy | INS |  |
| Paisey | 2003 | Alström Syndrome | PNS |  |
| Goodfellow | 2023 | Early versus late removal of urinary catheter after kidney transplantation | INS |  |
| Rydén A | 2020 | Nursing and training of pigs used in renal transplantation studies | Inclusion for full-tekst | PNS |
| Shimizu | 2021 | Predictive factors and management of urinary tract infections after kidney transplantation: a retrospective cohort study | Inclusion for full-tekst | INS |
| Bou Matar | 2012 | Routine transplant Doppler ultrasonography following pediatric kidney transplant | PNS |  |
| Capozza | 2010 | Renal transplantation in patients with "valve bladder": is bladder augmentation necessary? | INS |  |
| Adams | 2023 | Age-based risk of end-stage kidney disease in patients with myelomeningocele | PNS |  |
| Taghizadeh | 2007 | Ureterocystoplasty is safe and effective in patients awaiting renal transplantation | INS |  |
| Serrano | 1996 | Transplantation into the long-term defunctionalized bladder | PNS |  |
| Pirklbauer | 2022 | The Tangential Extraperitoneal Retrorenal Approach in Kidney Transplant Biopsy: An Observational Study to Assess Complication and Adequacy Rates | INS |  |
| Elkhammas | 1994 | Pancreas transplantation and dysuria | PNS |  |
| MacGregor | 1986 | Renal transplantation in end stage renal disease patients with existing urinary diversion | PNS |  |
| Kuiper | 2018 | Routine haemostasis testing before transplanted kidney biopsy: a cohort study | INS |  |
| Hatch | 1994 | Kidney transplantation in patients with an abnormal lower urinary tract | PNS |  |
| Davis | 1982 | Spontaneous bladder rupture and cytomegalovirus infection complicating renal transplantation: cause or coincidence? | INS |  |
| Liu | 2024 | Construction of a nomogram for predicting catheter-related bladder discomfort in patients with end-stage renal disease after renal transplantation: a retrospective study | INS |  |
| Ozdemir | 2019 | Nursing Care of the Patient With Neurogenic Bladder After Kidney Transplantation: A Case Report | Inclusion for full-tekst | PNS |
| Lapointe | 2001 | Urological complications after renal transplantation using ureteroureteral anastomosis in children | PNS |  |
| Antonio | 2022 | Treatment of asymptomatic bacteriuria in the first 2 months after kidney transplant: A controlled clinical trial | INS |  |
| Suaid | 2010 | Therapeutic option for infected urinary tract fistulas in renal transplantation | INS |  |
| Mittal | 2023 | Role of Intrathecal Morphine for Acute Postoperative Pain Management in Patients Undergoing Kidney Transplant: A Randomized Controlled Study | INS |  |
| Herbst | 2019 | Survival and Kidney Outcomes of Children with an Early Diagnosis of Posterior Urethral Valves | PNS |  |
| Sfakianakis | 2009 | A renal protocol for all ages and all indications: mercapto-acetyl-triglycine (MAG3) with simultaneous injection of furosemide (MAG3-F0): a 17-year experience | INS |  |
| Mokos | 2011 | Transurethral incision/resection of the prostate (TUIP/TURP) in operative treatment of repeated bladder outlet obstruction early after kidney transplantation | INS |  |
| Carrafiello | 2005 | The role of interventional radiology in the management of kidney transplant complications | INS |  |
| Neuhaus | 1998 | Bladder control a consequence of maturation: evidence after renal transplantation | Inclusion for full-tekst | PNS |
| Kamel | 2007 | Deceased-donor kidney transplantation in prune belly syndrome | PNS |  |
| Glazier | 1996 | Evaluation of voiding cystourethrography prior to renal transplantation | PNS |  |
| Biard | 2005 | Long-term outcomes in children treated by prenatal vesicoamniotic shunting for lower urinary tract obstruction | PNS |  |
| Qu | 2013 | [Combined application of 2 micron microwave resection and laparoscopy in treatment of urinary tumor in patients after renal transplantation] | INS |  |
| El-Arabi | 2020 | Cystoscopically Placed Pancreatic Duct Stent for Management of Bladder-Drained Pancreas Transplant with Pancreatic Duct Leak | INS |  |
| van Son | 1986 | Vesicocalicostomy as ultimate solution for recurrent urological complications after cadaveric renal transplantation in a patient with poor bladder function | INS |  |
| Svetanoff | 2021 | Neonatal Renal Failure in the Setting of Anorectal Malformation: A Case Report and Literature Review | PNS |  |
| Salvatierra | 1999 | A new, unique and simple method for ureteral implantation in kidney recipients with small, defunctionalized bladders | INS |  |
| Messing | 1985 | Bilateral rectus femoris pedicle flaps for detrusor augmentation in the prune belly syndrome | INS |  |

PNS: Population Not Suitable, INS: Intervention Not Suitable

**Supplemental file 2	Bladder training protocols**

1. Bladder training protocol 2019

Method

The catheter is removed in the morning. Afterwards, the patient must urinate every hour during the day. At night, the patient is woken up every two hours by the nurse to urinate.

This is followed by two bladder scans, for which the schedule below applies.

Bladder scan schedule

- < 100 ml: repeat scan after 4 hours;
- 100-200 ml: repeat scan after 3 hours;
- 200-300 ml: repeat scan after 2 hours;
- 300-400 ml: repeat scan after 1 hour;
- > 400 ml: consultation with the doctor on duty about one-time catheterization;
- > 700 ml: Consult with the doctor on duty about single catheterization or insertion of an indwelling catheter.

Note: If the measured volume is less than 100 ml twice, the check can be discontinued.

2. Bladder training protocol 2022

Method

According to the Clinical Care Pathway for Kidney Transplantation, the urinary catheter is removed on day 5 postoperatively by the nurse after consultation with the physician. To monitor the development of retention, a distinction is made between patients with preoperative urine output of more than 500 ml prior to kidney transplantation and patients with preoperative urine output of less than 500 ml prior to kidney transplantation.

> 500 ml preoperative urine output

After removal of the urinary catheter, the patient urinates every three hours during the day. The bladder scan is used twice to check for retention after urination. The patient is also observed to see if there is any inability to urinate. At night, the patient urinates when the urge arises. This is not followed by a bladder scan.

< 500 ml preoperative urine output

After removal of the urinary catheter, the patient urinates during the day when the urge arises (due to prolonged emptying of the bladder, overactivity and therefore incontinence is expected rather than retention). After removal of the urinary catheter, the bladder scan is used twice and the patient is observed to see if there is any inability to urinate. At night, the patient is woken up at 3 a.m. by the nurse to urinate. If the urge to urinate occurs earlier, the patient urinates at that time.

Bladder scan schedule

- 0-200 ml: repeat scan after 3 hours;
- 200-300 ml: repeat scan after 1 hour;
- 300-400 ml: consultation with the doctor on duty about one-time catheterization;
- > 400 ml: Consult with the doctor on duty about single catheterization or insertion of an indwelling catheter.

Note: If the measured volume is less than 100 ml twice, the check can be discontinued.

**Supplemental Material** **3	Results of regression analysis on interaction terms**

Table A	Univariate interaction terms

|  | **Urine Residual > 100 ml** | **Urinary Tract Infection** | **Re-catheterization** |
| --- | --- | --- | --- |
|  | **OR (95% CI)** | **OR (95% CI)** | **OR (95% CI)** |
|  | **Residual OR (CI)** | **UTI OR (CI)** | **Re-cath OR (CI)** |
| **Univariate with Interaction** |  |  |  |
| Post versus pre | 1.14 (0.54-2.42) | 0.76 (0.33-1.75) | 0.50 (0.16-1.60) |
| Age (centered at mean) | 1.01 (0.97- 1.04) | 1.00 (0.96-1.03) | 1.00 (0.96- 1.05) |
| Post versus pre * age centered | 1.03 (0.98-1.09) | **1.05** (0.99-1.12) | **1.09** (1.00-1.18) |
|  |  |  |  |
| Post versus pre | 1.49 (0.65- 3.46) | 1.11 (0.41- 2.98) | 0.98 (0.36- 2.70) |
| Female gender | 1.01 (0.32- 3.22) | 2.22 (0.73-6.81) | 0.82 (0.20-3.31) |
| Gender * post versus pre | 0.39 (0.07-2.21) | 0.58 (0.11-2.88) | 0.31 (0.02-3.86) |
|  |  |  |  |
| Post versus pre | 0.86 (0.30- 2.47) | 0.46 (0.15- 1.41) | 0.53 (0.13- 2.23) |
| Postmortal donor | 2.06 (0.69- 6.19) | 0.83 (0.24- 2.83) | 2.13 (0.60- 7.62) |
| Donor type * post versus pre | 1.55 (0.35-6.91) | 3.70 (0.69-19.97) | 1.60 (0.24-10.70) |
|  |  |  |  |
| Post versus pre | 1.10 (0.29- 4.12) | 0.36 (0.08- 1.62) | 1.48 (0.32- 6.87) |
| Preoperative up >500ml | 0.72 (0.22- 2.35) | 0.51 (0.16-1.60) | 1.02 (0.25- 4.15) |
| Preoperative up * post versus pre | 1.23 (0.25-6.04) | 3.41 (0.59-19.83) | 0.36 (0.05-2.47) |

Note: bold = p < 0.10
